# Biocontrol of *Fusarium graminearum* Growth and Deoxynivalenol Production in Wheat Kernels with Bacterial Antagonists 

**DOI:** 10.3390/ijerph110101094

**Published:** 2014-01-16

**Authors:** Cuijuan Shi, Peisheng Yan, Jiafei Li, Hanqi Wu, Qianwei Li, Shanshan Guan

**Affiliations:** School of Marine Science and Technology, Harbin Institute of Technology at Weihai, Weihai 264209, China; E-Mails: shicjwh@126.com (C.S.); lijfhit@126.com (J.L.); greenerysd@163.com (H.W.); xiangtuzi0607@126.com (Q.L.); guanshan654wh@sohu.com (S.G.)

**Keywords:** bacterial antagonists, biocontrol, deoxynivalenol, *Fusarium graminearum*, wheat kernels

## Abstract

*Fusarium graminearum* is the main causal pathogen affecting small-grain cereals, and it produces deoxynivalenol, a kind of mycotoxin, which displays a wide range of toxic effects in human and animals. Bacterial strains isolated from peanut shells were investigated for their activities against *F. graminearum* by dual-culture plate and tip-culture assays. Among them, twenty strains exhibited potent inhibition to the growth of *F. graminearum*, and the inhibition rates ranged from 41.41% to 54.55% in dual-culture plate assay and 92.70% to 100% in tip-culture assay. Furthermore, eighteen strains reduced the production of deoxynivalenol by 16.69% to 90.30% in the wheat kernels assay. Finally, the strains with the strongest inhibitory activity were identified by morphological, physiological, biochemical methods and also 16S rDNA and *gyr*A gene analysis as *Bacillus amyloliquefaciens*. The current study highlights the potential application of antagonistic microorganisms and their metabolites in the prevention of fungal growth and mycotoxin production in wheat kernels. As a biological strategy, it might avoid safety problems and nutrition loss which always caused by physical and chemical strategies.

## 1. Introduction

*Fusarium graminearum* Schwabe is the main causal pathogen of fusarium head blight (FHB) disease that affects small-grain cereals (wheat, barley and maize). FHB occurs throughout the World, causing serious problems for agriculture and the economy [1,2]. The fungus produces a number of mycotoxins during the process of growth and invasion of grains. Deoxynivalenol (DON) is the most common one. It’s the end product of the trichothecene biosynthetic pathway. As a potent protein synthesis inhibitor, DON causes a wide range of toxic effects in humans and animals [3,4,5].

Because of the hazards, a number of strategies have been developed to reduce the impact of FHB and mycotoxins, such as planting resistant varieties, use of appropriate fungicides, crop rotation, and harvesting timely and at low moisture content. Antagonistic microorganisms could also be effective for the inhibition of *F. graminearum* infections [6]. Some *Bacillus* strains and *Cryptococcus* strains could reduce the disease severity and increase 100-kernel weight of plants inoculated with *F. graminearum* [7]. *Paenibacillus polymyxa* exhibited potent inhibition to *F. graminearum* growth and DON production under greenhouse conditions [8]. Twenty-two bacterial strains, isolated from wheat anthers, have been proved to possess the ability to prevent FHB and DON production under greenhouse conditions. Nine strains significantly reduced both the disease severity and DON content in spikes, and five strains even decreased the mycotoxin to undetectable levels [9]. During the harvest period, moisture control and avoiding mechanical damage are also efficient strategies to prevent mycelia invasion and mycotoxin development [10].

The proper storage facilities for moisture and temperature control and aeration provide protection from mycotoxigenic fungal growth. Numerous natural and chemical agents have also been used to prevent the fungal growth and mycotoxin formation. Some studies have highlighted the potential use of antagonistic microorganisms to prevent the hazards of mouldy fungi particularly postharvest. For example, *Pichia anomala* was used to prevent spoilage by *Penicillium roqueforti* [11] and contamination of stored wheat by *P. verrucosum* and ochratoxin [12]. Laitila’s group reported that the cell-free extracts of two *Lactobacillus plantarum* strains were effective inhibitors to the growth of some *Fusarium* species in laboratory-scale malting of barley [13], but the effect of the two bacterial strains on the mycotoxin was not mentioned. Only a few reports have described DON inhibition in harvested grains by antagonistic microorganisms. Cheng *et al.* [14] obtained two *Bacillus* strains possessing the capability of detoxifying DON in wheat and maize contaminated by *Fusarium*. A U.S. patent documented that a bacterial isolate of *Bacillus* genus could transform DON in moldy corn to deepoxyvomitoxin (DOM), a less toxic product [15].

China is the largest producer of wheat in the World, and FHB is the epidemic disease in the country which causes *F. graminearum* contamination and mycotoxin formation during wheat storage [16]. *F. graminearum* always grows with high humidity which is hard to avoid during harvest and storage of grains. Physical and chemical strategies were applied to control FHB, but this might lead to safety problems and nutrition loss. Therefore, the aim of this study was to identify some bacterial strains with potential application in the prevention of fungal growth and mycotoxin formation in grains. Bacterial strains isolated from peanut shells were investigated for the ability to prevent *F. graminearum* mycelia growth *in vitro*. Meanwhile, inhibitory abilities of the selected strains on DON production in wheat kernels were tested. In addition, the bacterial strains with the highest activities against growth of mycelia and production of DON were identified.

## 2. Materials and Methods

### 2.1. Microorganism and Culture Media

The pathogen *F. graminearum* D5035, which produces the mycotoxin DON, was preserved at 4 °C on potato dextrose agar (PDA) slants (potato infusion from 20.0 g, 2.0 g of dextrose, 1.5 g of agar, in 100 mL of deionized water). To obtain the conidia suspension of *F. graminearum*, the mycelia were incubated in carboxymethylcellulose broth (7.5 g of sodium carboxymethylcellulose, 0.5 g of NH_4_NO_3_, 0.5 g of KH_2_PO_4_, 0.25 g of MgSO_4_·7H_2_O and 0.5 g of yeast extract in 1,000 mL of deionized water) for 7 days at 25 °C with shaking at 150 rev·min^−1^. The cultures were filtered through sterile gauze and the spore concentration was determined with hemacytometer and diluted to 2 × 10^5^ conidia·mL^−1^. The bacterial strains used for detecting antagonistic activity against *F. graminearum*, were previously isolated from peanut shells in our lab and stored at −20 °C in 30% glycerol-containing glucose and yeast extract (GY) broth (2.0 g of glucose, 0.5 g of yeast extract in 100 mL of deionized water) [17].

### 2.2. Antagonistic Activities against the Fungal Growth in Dual-Culture and Tip-Culture Assays

The antagonistic activities of the isolated bacterial strains against the pathogenic fungus were firstly investigated by a dual-culture plate method. The pathogenic *F. graminearum* was grown on PDA plates at 25 °C for 3 days, and then 4.0 mm diameter agar with mycelia from the plate was placed in the center of another PDA plate, in which four bacterial strains were inoculated one day ahead and 3.0 cm apart from the center of plate. The plates were incubated at 28 °C for 4 days and the diameters of *F. graminearum* growth were measured. Antifungal activities were expressed as the inhibition rate, (*r_c_* – *r*)⁄*r_c_* × 100% (*r_c_*: the radius of the *F. graminearum* without the presence of bacteria, *r*: the radius of the *F. graminearum* with the tested bacteria 3.0 cm apart from it).

The antagonistic activities were also studied with the bacterial cell-free culture supernatants by tip-culture assay according to Yabe *et al.* [18] with some modifications. The screened bacteria were cultured in GY broth at 35 °C on a rotary shaker at 150 rev·min^−1^. Four days later, the bacterial cells were removed by centrifugation at 7,155 *g* for 20 min. To maintain the uniformity of the growth conditions of *F. graminearum* in every tip, 2% glucose and 0.5% yeast extract which was the same as the GY broth were mixed into the cell-free supernatants, and the pH values were also adjusted similar to that of the GY broth. The supernatants were sterilized through 0.22 µm Millipore filter before use. Seven hundred µL of the cell-free filtrate and 10 µL of the conidia suspension of *F. graminearum* were mixed in a 5 mL tip tube with the bottom sealed with laboratory film (Bemis, Neenah, WI, USA). The tips were incubated at 28 °C for 6 days, and the mycelia were weighed.

### 2.3. Antagonistic Activities against Mycotoxin DON in Wheat Kernels Assay

The effect of bacterial cell-free culture supernatants on the DON production of *F. graminearum* was observed by wheat kernels DON assay according to Reddy *et al.* [19] with some modifications. About 10 g of healthy and dry wheat kernels (purchased locally, and the water content was 0.9%) were mixed with 2 mL of bacterial cell-free culture supernatants and 1 mL of conidia suspension in a 100 mL conical flask. In the control flask, bacterial culture supernatants were replaced by deionized water. For each bacterial strain and the control, there were three replicated flasks. After incubation at 25 °C for 18 days, the wheat kernels were ground by a grinder. Twenty-five mL of 84% methanol in water was added, and the mixture was shaken for 2 h, and then centrifuged at 7,155 *g* for 10 min. The solid residue was re-extracted, and the supernatants were combined. Before the analysis with HPLC, using Varian ProStar (Varian, Palo Alto, CA, USA) connected with UV-VIS detector (190–700 nm), the supernatant was filtered through 0.22 μm polyvinylidene fluoride (PVDF) syringe filter. The chromatographic column was a Kromasil C18 column (250 mm × 4.6 mm, 5 μm, Akzo Nobel, Bohus, Sweden). The mobile phase was 80% methanol in water and the flow rate was 0.8 mL·min^−1^, with the wavelength of detection at 218 nm. Peak area in the chromatogram was used to represent the relative DON content.

### 2.4. Identification of the Bacterial Strains

The identifications of the bacterial strains with high activities against the growth of mycelia and production of DON of *F. graminearum* were based on the preliminary morphological, physiological and biochemical analysis, and the further 16S rDNA and *gyr*A gene sequences analysis. The preliminary analysis included the colony morphology, Gram staining, cell morphology and size, spore production, growth at 50 and 65 °C, growth in sodium chloride of 4% and 7%, catalase test, V-P reaction, acid and gas production in glucose, nitrate reduction, starch hydrolysis test, casein decomposition test and lecithianse test.

For the gene sequences analysis, genomic DNA of bacteria was extracted with CTAB/NaCl method [20]. The primers for 16S rDNA PCR amplification were 5′-AGAGTTTGATCCTGGCTCAG-3’ and 5′-AAGGAGGTGATCCAGCCGCA-3′, and for *gyr*A gene amplification were 5′-CAGTCAGG AAATGCGTACGTCC-3′ and 5′-CAAGGTAATGCTCCAGGCATTG-3′. The 50 µL reaction mixture contained 5 µL of 10× buffer with MgCl_2_, 2 µL of template DNA, 2 µL of each primer (10 µmol·L^−1^), 5 µL of dNTPs (2.5 mmol·L^−1^ each) and 2 U of *Taq* polymerase. And the program was 94 °C for 5 min, 30 cycles of 94 °C for 30 s, 52 °C for 45 s and 72 °C for 90 s, and the final extension at 72 °C for 10 min. The PCR products were treated and analyzed by Sangon Biotech Co. Ltd. (Shanghai, China). The homology search of the sequences was performed with the blastn program in the web [21]. The neighbor-joining method with 1,000 bootstrap replications in program MEGA 5.05 was used to construct the phylogenic tree. The obtained nucleotide sequences of 16S rDNA for strains WPP9, WPP10, WPP1-2, XPP6-1 and WPS4-1 were submitted to Genbank and the accession numbers were KC422326, KC422327, KC422328, KC422329 and KC422330 respectively. Correspondingly, the accession numbers of *gyr*A gene sequences were from KC422331 to KC422335.

### 2.5. Statistical Analysis

All data were statistically analyzed by one-way analysis of variance using the SPSS 16.0 program (SPSS Inc., Chicago, IL, USA). Values were expressed as means ± standard deviation.

## 3. Results

### 3.1. Antagonism of Bacteria against Growth of F. graminearum in vitro

In the dual-culture plate test, all thirty-two of the bacterial strains showed antagonistic activity against the mycelia growth of *F. graminearum* after four days’ incubation, and the inhibition rates of 62.5% strains were more than 40%. The bacteria with the highest inhibition rate of about 55% were strains N_2_PS25-2 and WPP19-1. Some bacterial strains showed weaker inhibition activity, which was still significant difference with the negative control (*p* < 0.01) (Table 1).

**Table 1:** Antifungal activities of bacterial strains against *F. graminearum* growth in the dual-culture and tip-culture assays and against DON production in wheat kernels assay.

Bacterial Strains	Inhibition Rate of Mycelia (%) by Dual-Culture Tip-Culture		Inhibition Rate of DON (%) by Wheat Kernels Assay
N_2_PS25-2	54.55 ± 1.75	≈100 *^a^*	36.00 ± 3.04
WPP19-1	54.55 ± 0.00	≈100*^ a^*	30.49 ± 5.26
WPS2	52.53 ± 1.75	96.26 ± 0.57	27.80 ± 4.56
WPP17	52.53 ± 1.75	95.93 ± 0.51	30.49 ± 10.56
WPP10	51.52 ± 3.03	100	63.90 ± 2.00
WPP9	51.52 ± 3.03	100	88.40 ± 4.41
XPP6-1	51.52 ± 1.75	≈100*^ a^*	70.99 ± 3.40
WPS4-3	50.51 ± 1.75	≈100*^ a^*	56.00 ± 3.88
XPP6-2	49.49 ± 1.75	98.75 ± 0.85	26.80 ± 4.81
WPP14	48.48 ± 0.00	94.64 ± 0.74	20.12 ± 3.84
WPP19-2	47.47 ± 1.75	≈100*^ a^*	37.71 ± 2.57
XPP6-3	47.47 ± 1.75	96.93 ± 0.57	25.70 ± 10.70
N_1_PB1-2	47.47 ± 1.75	≈100*^ a^*	38.02 ± 3.60
WPS4-2	46.46 ± 1.75	95.40 ± 0.46	40.09 ± 2.64
WPP1-2	46.46 ± 1.75	97.84 ± 0.23	63.59 ± 3.08
HPP8	46.46 ± 1.75	92.70 ± 0.10	0
YPS8	45.45 ± 0.00	≈100*^ a^*	16.69 ± 4.28
WPP1-1	45.45 ± 0.00	97.44 ± 0.40	0
WPP19-3	44.44 ± 1.75	98.26 ± 0.79	32.00 ± 4.54
WPS4-1	41.41 ± 0.00	100	90.30 ± 1.01
SPS3	35.35 ± 1.75	–	–
N_2_PS9	33.33 ± 3.03	–	–
WPP16	32.32 ± 1.75	–	–
N_2_PS7	31.31 ± 1.75	–	–
N_1_PB1-1	28.28 ± 1.75	–	–
N_2_PS25-1	25.25 ± 1.75	–	–
WPP5-1	24.24 ± 0.00	–	–
WPP5-2	23.23 ± 1.75	–	–
SPP1	19.19 ± 1.75	–	–
WPP8-1	17.17 ± 1.75	–	–
WPG2	16.16 ± 1.75	–	–
WPP8-2	15.15 ± 0.00	–	–

Note: *^a^* The amount of mycelia was a little and weight was not quantified.

**Figure 1:**
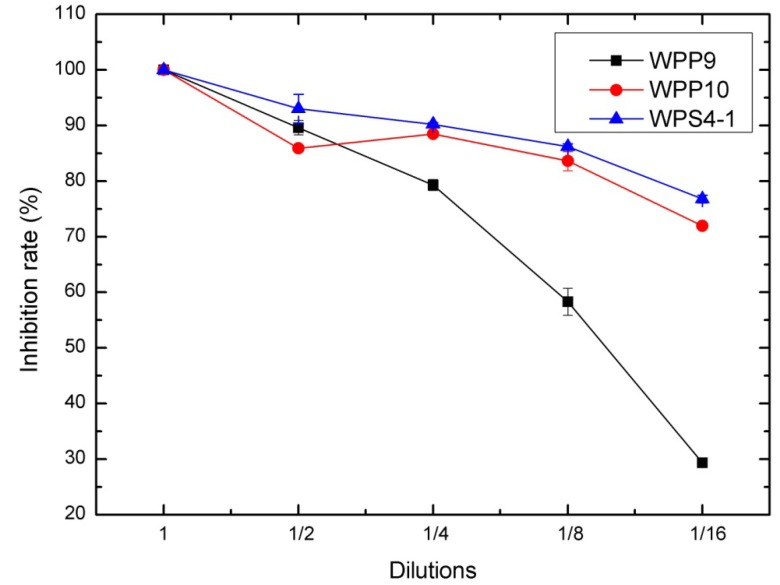
The inhibition of three bacterial culture supernatants with a serial dilution against *F. graminearum* growth in the tip-culture assay.

The antifungal activity of bacterial extracellular metabolites was studied by tip-culture assay using the cell-free culture supernatants. Almost all the strains, with inhibition rates of more than 40% in the dual-culture plate method, showed significant inhibition of the growth of *F. graminearum* (Table 1). The cell-free culture supernatants of WPP9, WPP10 and WPS4-1 could completely inhibit the germination of conidia. Furthermore, their culture supernatants with a serial dilution were used in tip-culture assay. The results showed that the inhibition rate was dose-dependent on the dilution multiples (Figure 1). Among them, strain WPS4-1 exhibited strongest inhibitory effect as it still inhibited the mycelia growth by 76.8% ± 0.7% even at 16-fold dilution. At the same time, strain WPP10 also exhibited an inhibition rate of 71.9% ± 0.4% at the same dilution rate.

### 3.2. Antagonism of Bacteria against DON Production of F. graminearum in Wheat Kernels

**Figure 2:**
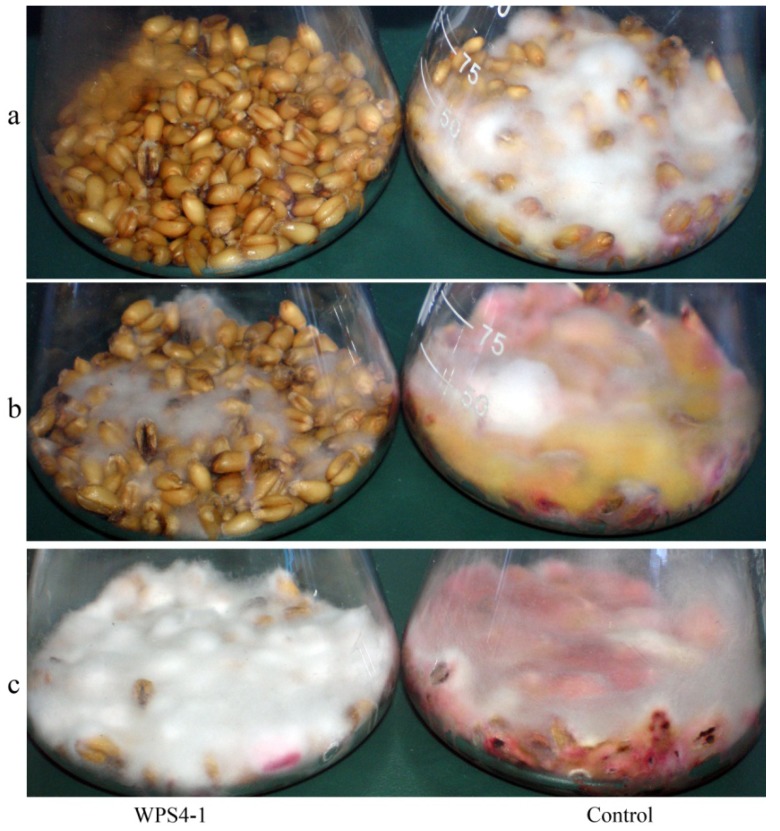
The mycelia growth of *F. graminearum* in wheat kernels with bacterial cell-free culture supernatant (strain WPS4-1) and deionized water (control) added. (**a**) four days of culture, (**b**) ten days of culture and (**c**) eighteen days of culture.

Based on the results of inhibition of *F. graminearum* growth, twenty bacterial strains were tested. After four days of incubation, there were some mycelia growing in the control wheat kernels with deionized water instead of the bacterial cell-free culture supernatants, while no mycelia were observed on the wheat kernels with the tested bacterial cell-free culture supernatants in the flasks (Figure 2a). On the tenth day, there were obvious differences in the antagonistic activities of the tested bacterial strains. In the flasks with the culture supernatants of some strains, such as WPP1-1 and HPP8, the inhibition activity was weak; whereas to some strains such as WPP10 and WPS4-1, the mycelia were obviously less than those in the control (Figure 2b). After 18 days’ of incubation, the wheat kernels were all covered with mycelia both in the control and tested flasks, and there were no differences of the amount of mycelia visibly (Figure 2c). The amount of DON produced in wheat kernels was determined by HPLC, and five strains XPP6-1, WPP1-2, WPP9, WPP10 and WPS4-1 with strong antagonistic activity against DON production were confirmed, with strain WPS4-1 showing the highest inhibition rate of 90.30% ± 1.01%, followed by strain WPP9 with an inhibition rate of 88.40% ± 4.41%. Strains WPP1-1 and HPP8 showed little inhibition activity towards the DON production in wheat kernels (Table 1).

### 3.3. Identification of Bacterial Strains with High Antifungal and Anti-Mycotoxin Activities

The selected bacterial strains XPP6-1, WPP1-2, WPP9, WPP10 and WPS4-1 showed similar characteristics in the morphological, physiological and biochemical analysis. They were Gram positive, round colonies with individual rods, and spore-producing bacteria. The cell size was (1.6–2.0) μm × (0.5–0.9) μm for strains XPP6-1, WPP1-2, WPP10 and WPS4-1, and (3.1–4.2) μm × (0.7–0.9) μm for strain WPP9. All the strains grew well in 4% or 7% sodium chloride, at 50 °C, but not at 65 °C. They could hydrolyze the tested substrates of starch, hydrogen peroxide, lecithin and casein, produce acid but not H_2_S in glucose, reduce nitrate to nitrite, and produce acetyl methyl carbinol in V-P reaction.

**Figure 3:**
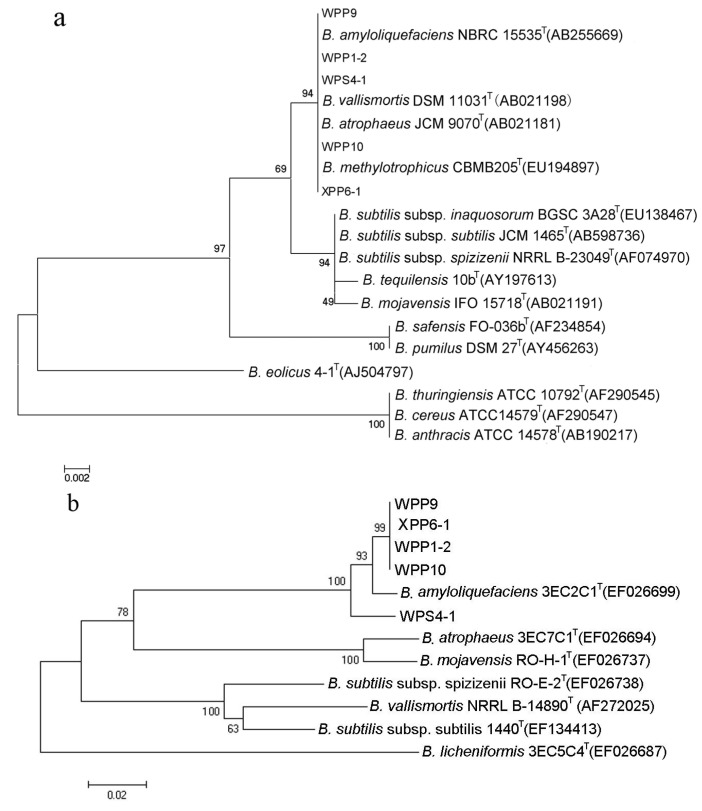
Phylogenetic trees of the selected five bacterial strains based on the (**a**) 16S rDNA and (**b**) *gyr*A gene sequences.

The homology search results of 16S rDNA sequences in GenBank database indicated that the five strains were similar to *Bacillus* sp., with the highest sequence similarity of 99%. All the strains were clustered into a group with the four standard *Bacillus* species in the phylogenetic tree (Figure 3a). The 16S rDNA sequences provided insufficient resolution to distinguish the close relatives of *Bacillus* species. Therefore, the housekeeping gene *gyr*A, effective for resolving these closely related taxa, was amplified and sequenced for the five selected bacteria. The phylogenetic tree based on *gyr*A gene revealed that the five strains were closely related to *B. amyloliquefaciens* (Figure 3b).

## 4. Discussion

Biological control is a promising strategy to control pathogenic fungi and mycotoxins. A large number of studies including *in vitro* dual-culture plate test, greenhouse and field trials were used to identify antagonistic microorganisms against mycotoxigenic fungi, such as *Aspergillus flavus*, *A*. *parasiticus* and *Fusarium* species [8,9,22,23]. The dual-culture plate assay has been used as a simple and fast method for preliminary screening of the antagonistic microorganisms *in vitro*. The mechanisms involved in control of FHB and DON production by microorganism have been proposed to be the production of antibiotics or competition for nutrients [9,24]. The present tip-culture assay tests of the antagonistic activity of the cell-free culture supernatants confirmed that the extracellular metabolites secreted by the bacterial strains could inhibit the growth of fungus as well as the production of mycotoxin.

FHB caused by *F*. *graminearum* occurs worldwide, and DON has a significant positive relationship with the aggressiveness of *F. graminearum*, leading to high contamination rates of *F*. *graminearum* and DON in crops. Many physical and chemical methods have been used for detoxification, such as heat treatment under alkaline conditions, extrusion cooking and hydrogen peroxide [25,26,27], but they are all restricted because of the safety problem, and loss of nutritional quality [28]. There are lots of studies related to antagonistic microorganisms against *F*. *graminearum* and DON focused on greenhouse and field trials, but there is little research about harvested agricultural products, [6]. Any potential biocontrol agent for grains must have the ability to reduce both fungal growth and DON production. Therefore, in the current study, the strains showing high antagonistic activity against fungal growth were further used for a wheat kernels assay. The growth of mycelia was observed for eighteen days and the DON content at the eighteenth day was measured, whether there was long-lasting inhibition activity of the bacterial culture supernatants should be further evaluated. In addition, the bacterial culture supernatants couldn’t completely inhibit the fungal growth and DON production, so they may be used together with some other agents or strategies in practice.

The results from the wheat kernels assay showed that all twenty selected bacterial strains could delay the germination of conidia in accordance with those seen in the *in vitro* dual-culture plate and tip-culture assays. Strains WPS4-1 and WPP9 showed the strongest DON production inhibition activity. Some strains, such as HPP8 and WPP1-1, showed different inhibition activity in the tip-culture and wheat kernel assays, which might result from their weaker or shorter-term activity. Currently, the mechanism for inhibition of mycotoxin production is not clear. Some thermosensitive substances in the culture supernatant can be responsible for the detoxification of DON [14]. Bakutis *et al.* [29] suggested that the detoxification of aflatoxins B1 and DON by *Saccharomyces cerevisae* was the contribution of glucomannans from the external cell wall. DON also could be converted to a much less toxic compound, or be adsorbed by microorganisms [15,30]. In the current study, it seemed that the inhibition of DON was due to the absence of fungal growth, but whether there was a direct inhibition effect needs further research.

According to the morphological, physiological and biochemical analysis, and the 16S rDNA and *gyr*A gene sequences analysis, the bacterial strains with high antagonistic activities were identified as *B. amyloliquefaciens*. Many studies have reported that *B. amyloliquefaciens* was a suitable antagonist against plant pathogens [24,31,32,33], and our results agreed with them. The selected strains could be potent antibiotics for biocontrol of *F. graminearum*. The mechanisms related to the reduction of DON production by antagonistic microorganisms will be further investigated. So far, the extracellular metabolites of *B. amyloliquefaciens* WPS4-1, playing the critical inhibition role, were identified as lipopeptide antibiotics of iturins according to the data from MALDI-TOF mass spectrometry (the authors’ unpublished data, list in references).

## 5. Conclusions

In this study, two *B. amyloliquefaciens* strains WPS4-1 and WPP9 were obtained showing reduction of DON production in wheat kernels by 90.30% ± 1.01% and 88.40% ± 4.41% based on the results of our established progressive screening methods of dual-culture, tip-culture and wheat kernels assays. Their extracellular metabolites played the critical role to the inhibitory ability. This study should provide a potential effective approach for the prevention of mycotoxin DON formation in wheat kernels, which is the use of extracellular metabolites from antagonistic microorganisms.
